# Impacts of Food Adulteration and Contamination: Health, Socio-Economic, and Legislative Aspects

**DOI:** 10.3390/foods15173041

**Published:** 2026-08-28

**Authors:** Mysha Momtaz, Saniya Yesmin Bubli, Mohidus Samad Khan

**Affiliations:** 1 Department of Chemical Engineering, Bangladesh University of Engineering and Technology (BUET), Dhaka 1000, Bangladesh; mm2855@njit.edu (M.M.); saniyayesmin.bubli@unh.edu (S.Y.B.); 2 Center of Materials for Advanced Energetics, New Jersey Institute of Technology, Newark, NJ 07103, USA; 3 Chemical Engineering and Bioengineering Department, University of New Hampshire, Durham, NH 03824, USA; 4 Institute of Appropriate Technology (IAT), Bangladesh University of Engineering and Technology (BUET), Dhaka 1000, Bangladesh

**Keywords:** food adulteration, food safety, intentional adulteration, incidental contamination, health impacts, regulations

## Abstract

Food adulteration is a critical global threat to public health, economic stability, and social welfare. It primarily occurs in two forms: intentional and incidental. Intentional adulteration, or economically motivated adulteration, involves deliberately adding fraudulent or toxic substances such as hazardous ripening agents and harmful coloring to increase profitability. Substitution of food species is also a widely practiced form. Such adulteration can cause severe acute and chronic health issues, ranging from gastrointestinal distress to cancer and organ failure. Incidental adulteration happens through accidental contamination during processing, storage, or distribution, involving hazards such as heavy metals, mycotoxins, and pesticides. Such contaminants also lead to severe chronic diseases and dangerous allergic reactions. Food adulteration also triggers massive socio-economic consequences. Food safety incidents such as the melamine milk crisis and the horsemeat scandal caused billions of dollars in losses due to healthcare costs, product recalls, damaged brand reputation, and disrupted international trade. Global entities such as the WHO, WTO, and Codex Alimentarius have established robust legislative frameworks. However, effective enforcement remains a significant challenge in many countries. To mitigate such harmful impacts, it is essential to strengthen global surveillance systems, enhance rapid analytical detection methods, and strictly enforce compliance with existing food safety regulations.

## 1. Introduction

Food is one of the basic needs of human beings, and thus food safety is considered the fundamental requirement for a food product. It has become a vital topic in recent decades for governments, food manufacturers, consumers, and the entire food supply chain. Food adulteration is the intentional or incidental addition of fraudulent or toxic substances to lower the quality of food. Food adulteration can also be identified by the substitution of food items with some inferior quality foreign particle or by the removal of some value-added food substitute from the primary food product. Intentional adulteration of food is also known as economically motivated adulteration (EMA) of food, which has a financial advantage [1,2]. Food products are also contaminated due to the lack of knowledge on proper hygiene at the time of processing, storage, transportation, and marketing [3]. However, any alteration in food quality may cause serious health-related problems for consumers if the adulterant is found to be toxic and allergenic [4].

Consumers judge food products based on pleasant feelings, such as taste, texture, odor, and visual appearance, when making a purchase. Hence, any abnormal aspects, such as discoloration, malodor, irregular proportions, or poor quality, negatively influence consumers’ choices. To satisfy customers’ demands, food products have become a source of adulteration to achieve economic benefits. The application of adulterants in the food sector has been growing since ancient times, largely due to limited legal controls on food quality and under-resourced authorities. Economically motivated adulteration of foodstuffs has been practiced for centuries, with special vulnerability in oil, flour, and beverages [5,6]. The first written account of food adulteration was published in the 19th century. Incidents such as cutting flour with alum and chalk to give a whiter appearance; tipping in plaster and sawdust to increase apparent weight; addition of strychnine to beer to give a bitter taste; and adding lead, copper, or mercury salts to increase the brightness of confectionery products were documented [7,8,9,10]. The report on wine adulteration by Cato was one of the oldest accounts of food adulteration. Fraudulent mixing of olive oil was a serious concern in Roman law at the time [11]. Accum, a pioneer of British consumer chemistry, described various types of food adulteration in a work that was published in 1820 [6,12]. Until the beginning of the 19th century, food production was carried out by small-scale producers with a high degree of personal accountability in marketing [11]. After that, traders and retailers began adopting fraudulent methods to gain a financial advantage in a competitive environment. Since then, food adulteration has become acute and caused serious health problems. Based on numerous reports of toxic adulterants [5,6], governments worldwide were compelled to enforce necessary laws, such as the Adulteration of Food Act, 1960, in England, and the Adulteration of Food Act, 1863, enacted by the State Parliament of Victoria [6]. The deceptive purpose of using adulterants leads to dangerous accidents from time to time. For example, the addition of melamine to milk in China caused six reported deaths, and about 300,000 Chinese infants and young children were injured [13]. Considering several harmful incidents and associated health hazards, food adulteration has become a rapidly growing global phenomenon that has garnered considerable public attention. On the other hand, incidental food contamination is also a long-observed phenomenon. It has been prevalent since ancient times, when foodstuffs were exposed to environmental contaminants. The 15th century saw the use of mercury, lead, and arsenic to control insect pests, as well as the development of copper-containing compounds to treat fungal diseases. Toxigenic fungi and mycotoxins entered the food chain around 10,000 years ago, when humans began cultivating crops and storing them for an entire season for the first time. During the 1960s, researchers reported the presence of chemicals, pesticides, veterinary drugs, and antibiotic residues in food [14]. The view of food safety began to change with outbreaks of human disease in the 1980s, driven by several bacterial contaminants and pathogens [15]. Today, in the modern food industry, incidental food contamination is a widely observed phenomenon.

Foods are often adulterated in several ways and are served to customers. Fish, fruits, and vegetables are often adulterated with chemicals and other preservatives to make them appear fresh. Bakery and confectionery products, milk, and beverages are adulterated using toxic compounds. Meat and meat products are substituted with other cheap, low-quality products, which can create religious violence, too. Moreover, coloring agents and artificial sweeteners are intentionally used to make quick profits. Fake food product advertisements that conceal product quality, incorrect labeling on food containers, and the sale of spoiled food are also associated with food adulteration. Additionally, food products can be contaminated by the improper use of pesticides during cultivation, the formation of biogenic amines during food processing, the accumulation of trace elements from the soil, the injection of antibiotics and veterinary drugs into animals during farming, microbial spoilage, and the invasion of mycotoxins and natural toxins, among other factors. In a nutshell, all kinds of foods and food products are contaminated in various ways, making it necessary to raise public awareness. Good health cannot be guaranteed solely by the health sector. It is also a civic responsibility to acquire knowledge about food contamination and be careful. Food contamination can cause mild to severe health diseases. Skin diseases, liver and stomach problems, kidney disorders, cancer, asthma, etc., can be manifested due to the intake of contaminated foods. Some immediate side effects, such as diarrhea, dysentery, and vomiting, may also occur after consuming poisonous food [16]. In 2011, the Centers for Disease Control and Prevention (CDC) reported that foodborne pathogens caused 48 million illnesses, 128,000 hospitalizations, and 3000 deaths worldwide [17]. Hence, a lack of knowledge about food handling and uncontrolled practice of food adulteration may negatively affect human health. Besides health impacts, food contamination negatively affects social and economic sectors. The United States alone incurs an annual economic loss of approximately $7 billion due to food safety incidents [18]. According to the Grocery Manufacturers Association, food fraud and adulteration may cost the global food industry between $10 billion and $15 billion per year, which affects approximately 10% of all commercially sold food items, and a company can face a cost of between 2% and 15% of annual revenue as a result of one incident [19,20]. A food fraud incident can result in long-term industry-wide losses, the loss of closed export markets, damage to valuable brands, and eroded consumer trust [20]. Public confidence and economic growth can be enhanced by addressing and preventing food adulteration risks more effectively. Consumers’ satisfaction depends on several factors, such as enhanced traceability, the use of local ingredients, accurate labeling, shorter supply chains, and information about product origin [20,21]. Different regulatory bodies aim to control the risks associated with food fraud and save the public from becoming victims of food adulteration.

This article systematically examines the common forms of food adulteration and contamination, health implications of adulterated food, the social and economic consequences of contamination incidents, and the regulations in place in various countries to monitor food adulteration and contamination. The scientific literature for the period of 2000 to 2026 was searched using the keywords “food adulteration”, “economically motivated adulteration”, “origin fraud”, ”incidental food contamination”, “health impacts of contaminated food”, “social impacts of food adulteration”, “economic impacts of food adulteration”, and “food regulations”. Approximately 250 articles were found to be more relevant and reviewed extensively. The Trello and European Commission food fraud databases, along with relevant newspapers and magazine articles, were reviewed for recent food adulteration incidents. This article aims to contribute to a deeper understanding of the health impacts associated with food safety and the current status of legislation on food quality monitoring.

## 2. Health Impacts of Intentional Adulteration

**Figure 1:**
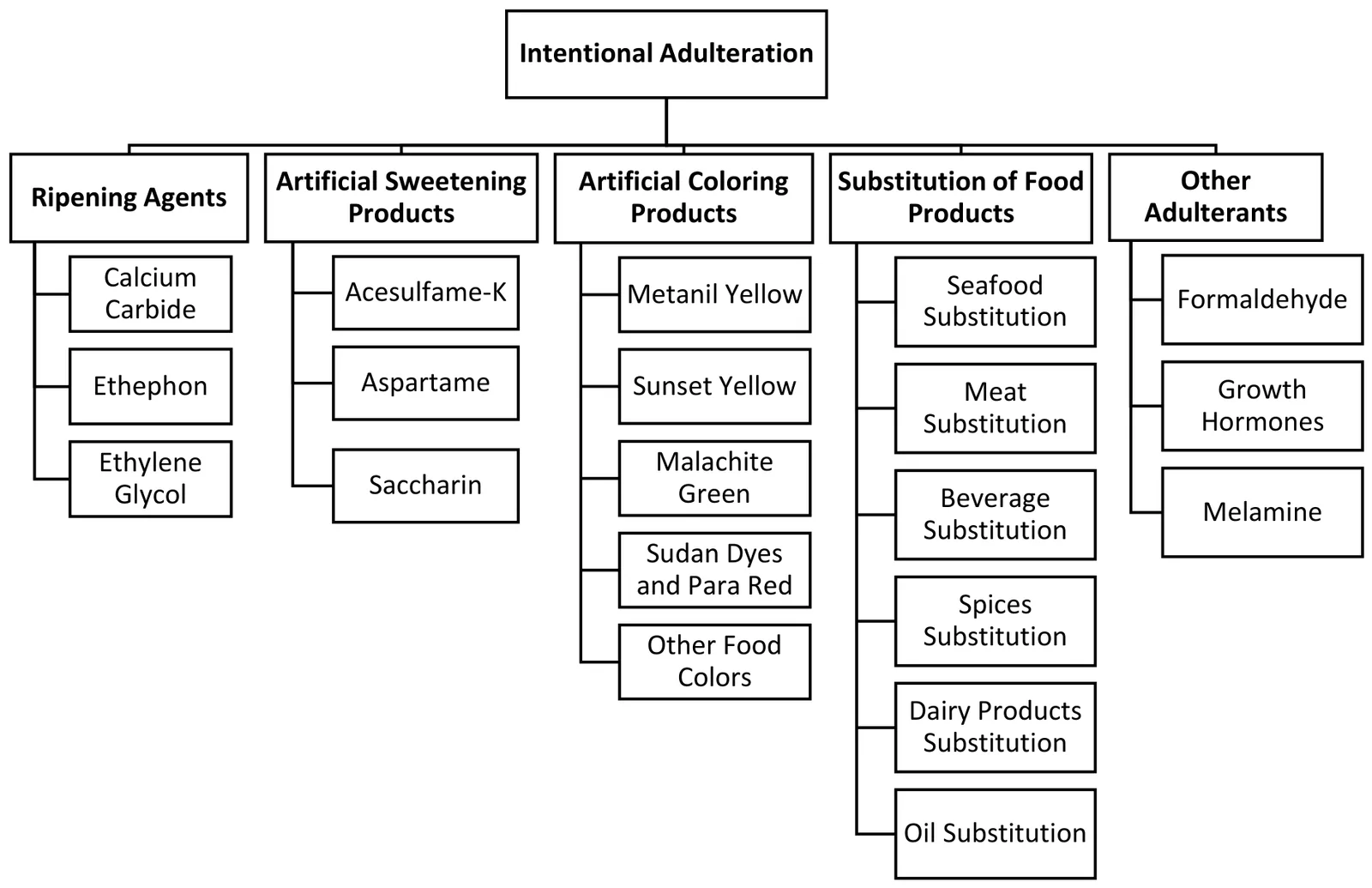
Intentional food adulteration with major categories.

Intentional or deliberate adulteration of food products encompasses all fraudulent practices intended to achieve economic gain. This type of practice involves substituting food products to increase the volume or weight of food samples. Moreover, adding dyes and harmful chemicals to maintain an attractive appearance and increase growth also resembles intentional adulteration [1,2]. For better understanding, intentional adulteration areas are divided into five major categories: ripening agents, artificial sweetening products, artificial coloring agents, food substitution with low-grade products, and other adulterants that have adverse impacts on human health. The categorization is shown in Figure 1 along with possible subclasses.

### 2.1. Ripening Agents

To meet the growing demand and satisfy economic factors, various types of artificial ripening agents are available worldwide. However, most ripening agents contain hazardous chemicals that cause significant acute and chronic health effects [22,23,24]. Ethylene, a naturally occurring ripening agent found in fruits, is a plant hormone that accelerates the ripening process [22,25]. Various chemicals are used to artificially mimic the ripening process of fruits and vegetables, which can reduce their nutritional value. Ethylene and methyl jasmonate are considered non-toxic artificial agents for human consumption, but these chemicals are costly [25,26]. Therefore, South Asian countries like Bangladesh, India, Pakistan, and Nepal use cheap ripening agents like calcium carbide [23,25], ethylene glycol [27], and ethephon [28].

#### 2.1.1. Calcium Carbide

Calcium carbide is a fast-acting, highly reactive ripening agent with carcinogenic properties [25]. When it comes into contact with moisture in the fruit containers, it produces acetylene gas as a ripening agent [22,25]. It is an alternative to ethylene and has an irksome odor. It lowers oxygen supply to the brain and affects the nervous system, causing headache, dizziness, sleepiness, mood disturbances, mental confusion, and seizures on a short-term basis, as well as memory loss and cerebral edema in the long term [22,25]. Commercial calcium carbide contains some impurities like arsenic and phosphorus hydride [29]. Hence, workers face serious health problems such as vomiting, dizziness, frequent thirst, irritation in the mouth and nose, burning sensation of the chest and abdomen, weakness, permanent skin damage, and difficulty in swallowing and speech [25,30]. Calcium carbide is alkaline in nature, and immediately after consumption, symptoms like abdominal pain, vomiting, and diarrhea can be observed [22].

#### 2.1.2. Ethephon

Ethephon is another ripening agent that accelerates ripening, resulting in fruits with a more suitable color. In the presence of moisture and at neutral pH, ethylene gas, bi-phosphate ion, and chloride ion are produced from the decomposition of ethephon, and these chemicals individually can be harmful to human health [25,26].

#### 2.1.3. Ethylene Glycol

Ethylene glycol is an odorless, colorless, and sweet-tasting liquid and is more commonly used as an antifreeze, coolant, and industrial solvent. It is inexpensive and can be diluted with water, making it cheaper and readily available. However, generous consumption of ethylene glycol can be very dangerous and may even cause kidney failure [27,31].

### 2.2. Artificial Sweetening Products

Due to the demand for sugar-free food, food manufacturers use different artificial sweeteners that contain low calorie content and imitate the effect of sugar on taste [32]. They are mainly preferable to consumers in four parts of the food and beverage markets—for obesity treatment, body weight maintenance, diabetes management, and prevention and reduction of tooth decay [33]. Sweeteners are natural or synthetic food additives that provide no or negligible nutritional value (“non-nutritive sweeteners”) with respect to the extent of sweetness [33]. The most common artificial sweeteners used in the food industry are aspartame, acesulfame-K, neotame, cyclamate, and alitame, but the breakdown products of these sweeteners may cause contentious health and metabolic effects [32].

#### 2.2.1. Acesulfame-K

Acesulfame-K has no calories and is 200 times sweeter than sucrose, also known as sugar. It can be used in baked goods, candies, frozen desserts, beverages, breath mints, and cough drops [34]. However, in very large doses, acesulfame-K can be clastogenic and genotoxic [34]. Moreover, one breakdown product of ace-K is acetoacetamide, which is known to be toxic if consumed at higher doses [32,35]. Sometimes, a headache can also be manifested as an acute symptom [34].

#### 2.2.2. Aspartame

Due to potential toxicity, aspartame has been considered the most controversial artificial sweetener [32,34]. It breaks down when exposed to prolonged heat. This property makes it unsuitable for baking or cooking. It can also decompose in liquids during storage [32]. Even with these limitations, aspartame is widely used as a sweetener in many low-calorie foods and drinks, including tabletop sweeteners, chewing gum, breakfast cereals, and other dry products [32]. Aspartame exposure may be responsible for headaches, dry mouth, nausea, vomiting, dizziness, reduced seizure threshold, and thrombocytopenia [34].

#### 2.2.3. Saccharin

Saccharin is the first artificial sweetener and is commercially used in a variety of products, including baked goods, soft drinks, tabletop sweeteners, jams, and chewing gum [34]. There is evidence of DNA damage due to saccharin [36]. Damage to genetic material, chromosomal aberrations, and exchange of sister chromatids are noticeable risk factors because of this particular sweetener [36]. Some studies have shown a relationship between saccharin consumption and cancer rates [32]. In other cases, sometimes saccharin intake can cause nausea, vomiting, and diarrhea [34].

### 2.3. Artificial Coloring Products

Artificial color additives are used to enhance the appearance of foods and pose a constant threat to both the food industry and human health. In some cases, colorants are used to grab children’s attention, thereby increasing the products’ sales. Both natural and synthetic colorants are used to meet consumers’ demand; however, synthetic dyes have more considerable applications due to high light stability, high availability, and lower production costs [37].

#### 2.3.1. Metanil Yellow

Metanil yellow (MY) is a yellow azo dye with wide applications in various industries, including coloring wool, nylon, silk, paper, ink, aluminum, and detergents [38]. However, this colorant is not permitted for use in food materials because it is toxic. Despite being banned for use in foodstuffs, this toxic chemical is widely used in various food products due to its low cost. In developing countries, it is used in sweat meat, ice-creams, soft drinks, turmeric powder, and beverages [39]. Metanil yellow, a toxic colorant, may cause damage to the heart, liver, kidneys, intestines, gastric tissue, nervous tissue, etc., all necessary organs and organ systems of the human body [38]. Methanol yellow, a common variation of Metanil yellow, is also used in melons and watermelons to boost the color and appearance. It can lead to stomach diseases, cancer, and deterioration of male reproductive organs [36].

#### 2.3.2. Malachite Green

Malachite green (MG) is widely used as a parasiticide in the aquaculture industry. It is highly effective against important protozoal and fungal infections, as well as certain helminth-caused diseases, in various fish and other aquatic organisms [40,41]. On the other hand, it is also used in various industries, including food, health, textiles, and others, for multiple purposes. In the food industry, it is commonly referred to as a food coloring agent or food additive. However, malachite green has been considered highly controversial because it can pose a risk to consumers of treated fish. Although the dye is not approved by the U.S. Food and Drug Administration and has been banned for use in various countries, its application is still observable in many parts of the world due to its low price and widespread availability [41]. The dye is toxic and can have adverse effects on the immune and reproductive systems, as well as exhibit genotoxic and carcinogenic properties [41,42,43]. Histopathological effects of MG are detrimental and involve multi-organ tissue injury [41]. In addition, the reduced form of MG is leucomalachite green, which is found in both aquatic and terrestrial ecosystems as a contaminant and poses a possible human health hazard [41].

#### 2.3.3. Sunset Yellow

Sunset yellow (SY) is a synthetic yellow azo dye widely used in various food items, including sweets, jams and jellies, soft drinks, candies, canned juice, sauces, pickles, and ice cream [44,45]. However, this dye was banned in the United States and Japan due to mutagenicity evidence found in several mammal bioassays [45]. Moreover, this dye may cause an allergic reaction in people with an aspirin intolerance, which may result in different symptoms, including gastric upset, vomiting, diarrhea, migraines, nettle rash (urticaria), and swelling of the skin (angioedema) [44]. It is also reported that SY has been linked to hyperactivity in children [46]. On the other hand, the effect of sunset yellow is not identical for every individual; rather, it can vary according to dose, age, gender, genetic factors, nutritional status, and time of exposure [44,45].

#### 2.3.4. Sudan Dyes and Para Red

Sudan dyes are a class of synthetic azo dyes and organic colorants generally used in industrial or printing products [47,48,49,50]. Para Red (PR) and Sudan dyes (Sudan I, II, III, IV, and Sudan G) as food colorants have been confirmed to be carcinogenic to human health [47,48]. Recently, it has been reported that some common species of human intestinal bacteria can reduce Sudan dyes (Sudan I, II, III, and IV) and PR into potential carcinogenic aromatic amines [50,51]. Sudan I is a known mammalian carcinogen [52] and is also a powerful contact allergen and sensitizer, causing pigmented contact dermatitis in humans [53,54]. Sudan IV becomes mutagenic after chemical reduction and metabolic activation [52]. Introduction of Sudan dyes and Para Red in humans can occur through ingestion, inhalation, or skin contact [50,51]. These dyes can pose a potential health hazard to humans by contaminating the food chain. They are widely used as food contaminants in seafood, hot chili, other spices, baked foods, and eggs to improve and maintain the appearance of food products [47,55,56,57,58]. The widespread use of these dyes can lead to significant exposure.

#### 2.3.5. Other Food Colors

Some food colors have detrimental impacts on consumer health. For example, hyperactivity may be initiated in children due to the consumption of food containing common food dyes like Yellow 2 and Red 40 [36]. Yellow 5, used in candies, cereals, and baked products, is strongly associated with hypersensitivity, hyperactivity, and other behavioral effects [36]. Blue 2 in confectionery, candies, pet foods, beverages, etc., may result in brain tumors [36]. Other examples of malpractices are the use of copper sulfate in green vegetables such as bitter gourd and ladyfingers, which may result in anemia due to prolonged consumption, and the use of lead chromate in melons and watermelons, which may result in brain injury, blindness, and anemia [36]. In addition, Orange II and Rhodamine B are also found in some spices, showing genotoxic and carcinogenic properties [20,59]. Sometimes, dyes and meat-flavor essences are added to spoiled beef meat to cover the changed color and unpleasant odor, but the toxic elements produced by bacterial metabolism are still present, which will put human health in danger [60].

### 2.4. Substitution of Food Products and Related Health Hazards

Mislabeling, fraud, and substitution with cheaper products are common fraudulent practices of food adulteration. These will not only negatively affect human health but also sometimes violate people’s religious beliefs.

#### 2.4.1. Seafood Substitution

Seafood substitution is responsible for exposure to allergenic foods and to fish with high toxicity, as well as to contaminated substituted species [61]. For example, pufferfish (*Lagocephalus scleratus*), known to cause tetrodotoxin poisonings, is often falsely labeled as monkfish so that production costs can be lowered, and import and other restrictions can be avoided [19]. Paralysis of the respiratory muscles and a significant number of consumer deaths are reported due to this misbranded pufferfish. More than half (58%) of substituted samples for other species are considered unsafe for human health [61].

#### 2.4.2. Meat Substitution

There is evidence of beef-containing foods adulterated with horse meat. Horse meat itself does not cause a health risk, but illegal entry of horse meat containing phenylbutazone (a veterinary drug) into the food chain has detrimental effects [62]. Consumption of food products contaminated with pork or its derivatives may cause allergic reactions [63,64]. In addition, chronic diseases like diabetes and cardiovascular disease can result from the the accumulation of cholesterol and saturated fats in the human body following high levels of consumption of pork-adulterated foods [64,65].

#### 2.4.3. Beverage Substitution

The nutritional value of fruit juices is often reduced by adulteration. Some substituted ingredients can be toxic and may lead to allergic reactions in humans [66]. Serious and potentially harmful pharmacological interactions can occur when orange juice is partially replaced with cheaper grapefruit juice. Grapefruit juice contains naringin, the major flavonoid glycoside, which is metabolized to the flavonone naringenin in the consumer’s body and affects the clinical modulation of drug transport. As a result, bioavailability gets altered and eventually results in serious consequences [67,68]. There is evidence that a Turkish traditional aniseed-flavored distilled spirit, which is known as Raki, has been adulterated with methanol, resulting in severe health problems, even death [68].

#### 2.4.4. Spices Substitution

Direct adulteration of spices (cumin, paprika) with nut proteins is a matter of serious public health concern, with evidence that they cause anaphylaxis [69]. Cumin is also adulterated with poor-grade fennel seeds coated with waste marble dust and dyes, posing potential health hazards [20]. It is reported that due to a similar appearance, adulteration of Chinese star anise (*Illicium verum*) with Japanese star anise (*Illicium anisatum*) causes intoxication in children. As it contains potent neurotoxins, Japanese star anise is responsible for neurological and gastrointestinal problems in children [70]. Adulteration of black pepper with papaya seeds can result in liver and stomach problems [20]. Turmeric has been adulterated with yellow chalk powder (a cheap material), and this fraudulent practice has several detrimental effects, such as swelling of the face, loss of appetite, nausea, and vomiting [71]. Turmeric is also reported to be adulterated with *Curcuma zedoaria*, having toxic effects in rats and chickens [20,72]. Another adulteration of turmeric involves the use of lead chromate as a dye and a bulking agent. Delayed mental and physical development can be caused by exposure [20]. The fraudulent practice of adding olive leaves to oregano can pose a risk to human health [73].

#### 2.4.5. Dairy Products Substitution

There is evidence that most milk proteins, even with low concentrations, are known to cause food allergies. Some people are allergic to cow’s milk, and caprine milk, being easily digestible and low in lactose, is a good alternative. Therefore, consuming ovine or caprine milk adulterated with bovine milk or whey can have negative impacts [74,75,76,77,78]. Bovine milk is also found to be used in non-bovine cheese-making [78].

#### 2.4.6. Oil Substitution

Accidental or intentional adulteration of mustard oil with argemone oil and butter yellow may cause gallbladder cancer [79]. Moreover, argemone oil mixed with edible oils can result in epidemic dropsy, glaucoma, and loss of eyesight [80,81]. It has been reported that clinical symptoms of argemone oil poisoning include vomiting, diarrhea, nausea, swelling of limbs, erythema, pitting edema, breathlessness, etc., and glaucoma, and even death due to cardiac arrest in extreme situations [81]. Two physiologically active alkaloids, sanguinarine and dihydrosanguinarine, are characterized as the toxic compounds present in argemone oil [80,81].

Due to the high price of cold-pressed oil, admixing it with refined oil is a common fraudulent scenario nowadays. Cold-pressed oil is in high demand because it is not in contact with chemicals and solvents, and some minor compounds are also well-preserved. However, trans fatty acids and steradienes are formed during refining processes, which are normally absent in cold-pressed oil. Serving no useful purpose, these trans fatty acids do not promote good health and can increase the risk of coronary heart disease. In addition, trans fats produced from partially hydrogenated oils are considered more harmful than naturally occurring oils [80,82]. On the other hand, the risk of cancer, paralysis, liver damage, and cardiac arrest has been attributed to heavily adulterated loose edible oil [80].

Edible oil contaminated with castor oil may lead to stomach problems, and edible oil adulterated with mineral oil can cause damage to the liver, as well as produce carcinogenic effects [83]. In addition, there is evidence of more than 20,000 people being affected by olive oil spiked with aniline-denatured rapeseed oil [84]. Spanish toxic oil syndrome, or Spanish olive oil syndrome, resulting from selling non-edible rapeseed oil as edible rapeseed oil, and even as olive oil, has been reported to cause serious health hazards [82].

### 2.5. Other Adulterants

Besides the above-mentioned adulterants, other deliberate adulterants enter our food chain and cause irreparable damage to human health. Some common adulterants intentionally added to many regular foodstuffs to increase profits are described below.

#### 2.5.1. Formaldehyde (FA)

At normal temperature and pressure, formaldehyde is a highly reactive, flammable, and readily polymerizing colorless gas [85]. An aqueous solution of formaldehyde (37–40 wt%) is known as formalin, which is a colorless liquid and used as a biological preservative [85,86]. Formaldehyde is widely used for various purposes, and its application as an artificial preservative has been found in fish, fruits, vegetables, drinks, sweetmeat, milk, cheese, ice cream, and spices [86,87,88]. Formaldehyde is a naturally occurring substance in food, and it can complicate the detection of artificially added formaldehyde. Formaldehyde exposure can occur through inhalation of its gaseous form or by ingestion of substances [87]. Dermal absorption may be another possible route for formaldehyde consumption [87].

FA has detrimental impacts on the nervous system, kidneys, liver, and lungs, and may cause asthma, pulmonary damage, abdominal pain, vomiting, nausea, gastrointestinal lesions, and cancer [86,88,89]. It might be related to nasopharyngeal cancer, gastrointestinal cancer, and leukemia through inhalation [86,88,90]. Acute formaldehyde exposure may cause irritation to different body parts (eyes, nose, throat, and skin) [87]. FA is also responsible for metabolic acidosis, proteinuria, hematuria, tachypnoea, jaundice, reduced blood cells, and acute renal failure [87]. DNA and chromosomal damage can also result from formaldehyde exposure [87].

#### 2.5.2. Growth Hormones

Several growth hormones are used to promote growth and enhance the color of vegetable crops. Chemicals such as gibberellic acid, alpha-naphthylacetic acid, copper sulfate, and oxytocin are commonly used by many farmers in vegetable production. Among these, oxytocin is a mammalian hormone used as a veterinary drug and is unsuitable for use in fruits and vegetables, such as bottle gourds, bitter gourds, pumpkins, and cucumbers. Some detrimental impacts due to prolonged consumption of these hormones can be uterine cancer, exhaustion and loss of energy, early or irregular periods in women, balding in men, etc. [36].

#### 2.5.3. Melamine

Nitrogen-rich compounds can mimic high protein concentration, and in this case, standard methods cannot differentiate nitrogen derived from protein sources from that derived from non-protein sources [91]. Melamine (1,3,5-triazine-2,4,6-triamine, C_3_H_6_N_6_), a nitrogen-rich (66.6% nitrogen by mass) organic compound [92,93], is fraudulently used to adulterate milk, food, and feed materials [91]. Consumption of melamine above the safety limit can cause renal failure, kidney stones, urinary tract effects, and death in infants, who are more dependent on milk-based products [91,93,94].

All possible intentional adulterants and associated health hazards are summarized in Table 1, along with their potential sources.

## 3. Health Impacts of Incidental Food Contamination

**Figure 2:**
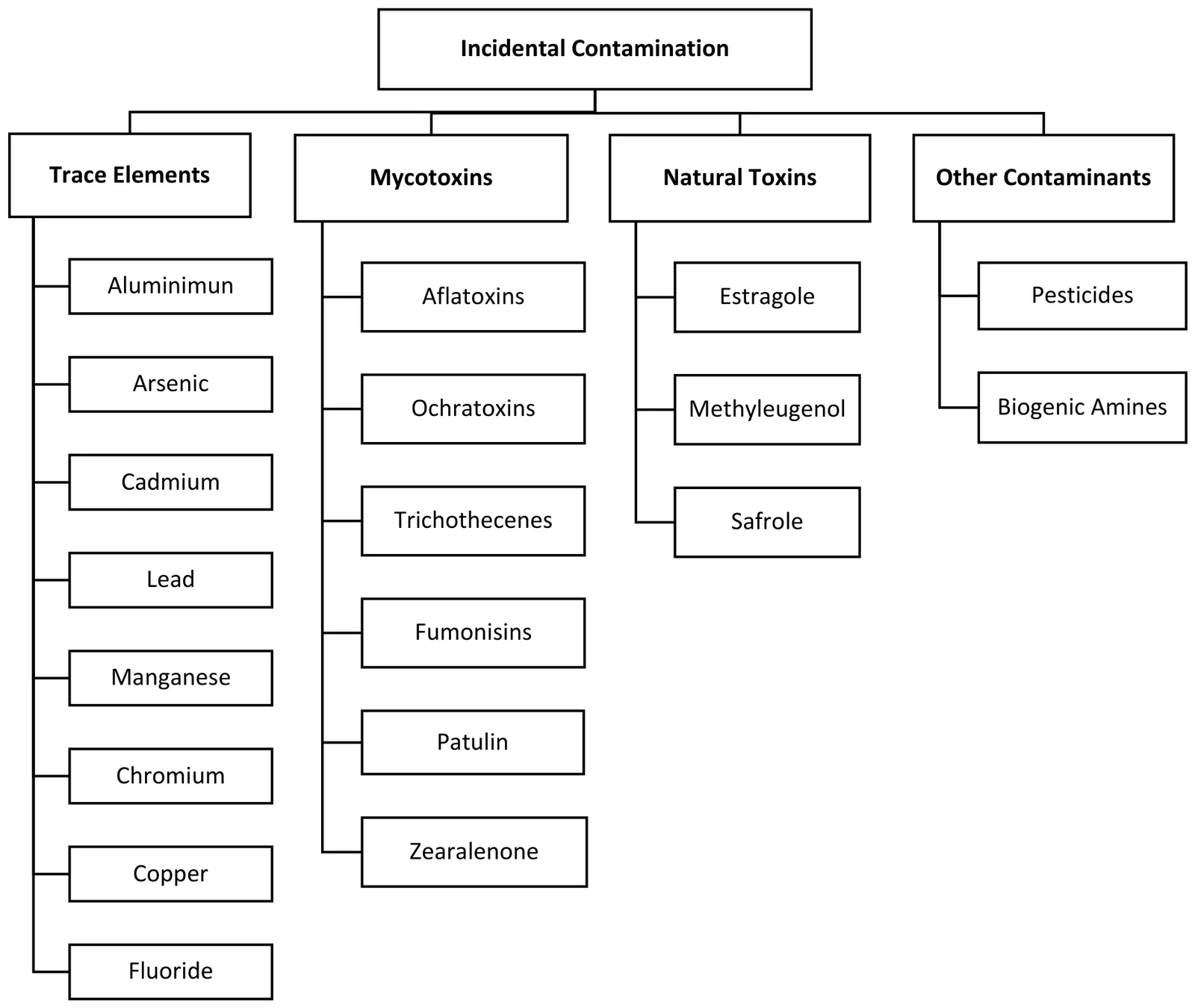
Incidental food contamination with major categories.

Incidental food contamination involves food quality degradation due to a lack of knowledge about handling foodstuffs and accidental contamination caused by environmental or biological issues. It can occur during food processing, packaging, and storage. Sometimes, inherent contamination of food (presence of organic compounds, some chemicals, or radicals naturally occurring in food) may cause food quality degradation [16]. Incidental food contamination can be divided into four categories, which can pose harmful effects on human health. The classification is presented systematically in Figure 2.

### 3.1. Trace Elements

Heavy metal contamination is very harmful to the ecological community due to the persistence of these contaminants in the environment [96]. Regarding trace elements, it has been estimated that 93% of them enter the human body through solid food consumption, and only 7% come from liquids [97]. Apart from the vital macro- and microelements, some trace elements with a high relative atomic mass are also found in tea leaves. Excessive consumption of these unnecessary trace metals can increase the body burden in humans over time [98]. Tea plants are commonly grown in highly acidic soils, where trace elements are potentially more bioavailable for root uptake, which is the reason for the presence of these metals in tea plants [99]. Some trace elements and associated health risks are described below.

#### 3.1.1. Aluminum (Al)

A major problem in agricultural plants is aluminum toxicity [100]. Aluminum (Al) is a very strong neurotoxicant and also causes brain disease, softening of bones, and anemia, especially in patients with chronic renal failure on dialysis [100,101]. However, such effects can also be seen in patients without kidney failure [100,101]. Significant evidence suggests that aluminum may be responsible for the pathogenesis of Alzheimer’s disease (AD); however, it is still a controversial issue whether the connection is causal [101,102]. Hence, a high Al concentration in food materials is a matter of concern.

#### 3.1.2. Arsenic (As)

Human exposure to arsenic (As) can occur through air, food, and water [103]. It is a naturally occurring element that is highly toxic to plants, animals, and human health. Consumption of arsenic-contaminated drinking water results in blackfoot disease [104,105]. People are at risk of lung and skin cancer, and this element may cause other internal cancers as well [104]. Skin lesions (characterized by symptoms like hyperpigmentation and keratosis), peripheral neuropathy, and anemia are strongly associated with chronic arsenic ingestion [104,105].

#### 3.1.3. Cadmium (Cd)

Agricultural and industrial activities have been identified as the main drivers of the widespread distribution of cadmium (Cd) into the environment and the human food cycle [106,107]. Soft water contains more Cd than hard water [97]. Cadmium is also carcinogenic and has been related to lung and prostate cancer [98,104]. Cognitive development and intellectual performance in children can be impaired by cadmium, which can also affect the kidneys and reproductive system [104]. Hypertension due to Cd exposure has been confirmed in laboratory animals and in humans, and blood pressure is also increased by Cd [97]. This metal has been strongly linked with renal toxicity [106] and is also a major risk factor for phytotoxicity [98].

#### 3.1.4. Lead (Pb)

Lead (Pb) is among the most commonly occurring toxic substances in the environment. Food and beverages are among the principal sources of Pb found in the human body [98]. Pb can enter the food cycle through the environment (for example, uptake by plant roots or foliage) or from food processing and storage [99]. Lead is a physiologic and neurological toxin and has the potential to affect many organs and organ systems in the human body [99]. The central nervous system is the most sensitive part of the body, especially in children. Lead at low concentrations can reduce cognitive development and intellectual performance in children, as well as damage the kidneys and the reproductive system [99,104]. Serum Pb is also responsible for hypertension activity [97].

#### 3.1.5. Manganese (Mn)

Both inadequacy and excessive exposure to manganese can create severe effects. This element is responsible for neurological effects following inhalation exposure, especially in occupational settings [98].

#### 3.1.6. Chromium (Cr)

Chromium (Cr) is an essential trace element; however, when it enters the human food chain, it can cause harmful effects. Cr has wide applications in metals and chemical industries, and it occurs in various forms in the environment [108]. Cr is introduced into the environment, especially in trivalent [Cr (III)] and hexavalent [Cr (VI)] forms, due to natural processes and human activities [108]. Cr (VI) is a strong oxidant and deleterious environmental pollutant and is more toxic to biota than Cr (III) [98,108]. Cr (VI) is carcinogenic and genotoxic and has the potential to pollute soil, surface water, and groundwater [108]. Moreover, kidney and liver damage, skin lesions, or rashes in human beings are results of chromium toxicity [108]. Cr also has harmful effects on microbes [108].

#### 3.1.7. Copper (Cu)

Copper (Cu) is an essential nutrient for human metabolism; however, excessive intake of Cu from food and water is detrimental [109,110]. Ingestion of contaminated food products, beverages, and drinking water, or intentional or unintentional consumption of high amounts of copper salts, leads to acute copper toxicity, which is rare in humans [111]. Excessive salivation, epigastric pain, diarrhea, nausea, and vomiting are the manifestations of acute poisoning [111]. Severe manifestations of copper toxicity, such as intravascular hemolytic anemia, acute tubular renal failure, acute hepatic failure, coma, shock, and death, has been noticed [111]. There are also informal reports of acute gastrointestinal symptoms, including nausea, vomiting, diarrhea, and abdominal pain, in humans [110,111].

#### 3.1.8. Fluoride

Dental and skeletal fluorosis can result from the dietary intake of high quantities of fluoride [112]. Normally, drinking water is the major source of fluoride [112,113], but brick tea ingestion also causes outbreaks of fluorosis [112].

### 3.2. Mycotoxins

Mycotoxins are natural contaminants of cereals and other food products, and they are toxic secondary metabolites produced by filamentous fungi. Exposure to mycotoxins through consumption, ingestion, inhalation, or absorption through the skin may cause several acute and chronic human diseases. The three most important genera of mycotoxigenic fungi are *Aspergillus*, *Fusarium*, and *Penicillium*, and they frequently occur in major food crops, including cereals, peanuts (groundnuts), and various fruits, before and after harvest. The major classes produced by these genera are aflatoxins, ochratoxins, trichothecenes, and fumonisins [114,115].

#### 3.2.1. Aflatoxins

Aflatoxins are found in maize, peanuts, wheat, rice, tree nuts, and dried fruits [114,115]. The two major *Aspergillus* species that produce aflatoxins are *A. flavus* and *A. parasiticus* [114,116]. Aflatoxins are acutely toxic, immunosuppressive, teratogenic, mutagenic, and carcinogenic [116]. The primary target organ for carcinogenicity and toxicity is the liver [116]. Several aflatoxicosis outbreaks have occurred in hot, humid regions worldwide.

#### 3.2.2. Ochratoxin A

Ochratoxins are known as secondary metabolites of *Aspergillus* and *Penicillium* species [117]. They are found in cereals, coffee, bread, and on various food items of animal origin in many countries [116]. The most common ochratoxin is ochratoxin A, which is the most toxic [116,117]. Ochratoxin A occurs mainly in cereals, but considerable amounts of contamination may also be found in wine, coffee, spices, and dried fruits [115]. It can be carcinogenic, teratogenic, immunosuppressive, nephrotoxic, and may also cause urinary tract tumors [115,116].

#### 3.2.3. Trichothecenes

Most members of the *Fusarium* group produce trichothecenes, although other genera like *Trichoderma, Trichothecium, Myrothecium, and Stachybotrys* are also known to produce these mycotoxins. Among 148 isolated trichothecenes, only a few have been discovered to contaminate food and feed. The major contaminants are deoxynivalenol (DON), nivalenol (NIV), and diacetoxyscirpenol (DAS), while T-2 toxin is uncommon. Depression of immune responses, nausea, and vomiting are some common symptoms of trichothecene toxicity [116].

#### 3.2.4. Fumonisins

Fumonisins and mycotoxins are produced by *Fusarium moniliforme* and related species. Fumonisins B_1_ and B_2_ are toxic, while the others (B_3_, B_4_, A_1,_ and A_2_) are less toxic and occur in very low concentrations [116]. Maize and maize-based products are the main sources of fumonisins. Fumonisins can exhibit carcinogenicity [115].

#### 3.2.5. Patulin

Patulin is a secondary mold metabolite of different types of *Penicillium* and *Aspergillus* species [118]. Nowadays, *Penicillium expansum*, commonly known as the blue mold, is known as a major source of patulin contamination. This fungus causes soft rot in apples, pears, cherries, and many other fruits. Patulin is often present in unfermented apple juice. Low-acid fruit juices, especially apple, grape, and pear juices, are most often contaminated because these fruits are commonly infected by fungi that produce patulin. Patulin contamination can harm the stomach and intestines, the respiratory system, DNA, and several important enzymes [114].

#### 3.2.6. Zearalenone

Zearalenone is mostly found in wheat and maize, but also in sorghum, barley, and compounded feeds [116]. It is produced by *Fusarium graminearum* and *Fusarium culmorum* species [114]. Zearalenone and its derivatives can be associated with estrogenic effects in many animal species [116]. Premature puberty in girls and cervical cancer have been associated with zearalenone contamination [114].

### 3.3. Natural Toxins

To meet the increasing market demand for food products with “real health benefits”, natural ingredients have attracted customers. Therefore, consumers’ preference for botanical products as bioactive constituents in functional foods, as food supplements, and as herbal teas and food flavors has developed. However, botanical or herb-based preparations may contain components known to be toxic, genotoxic, and carcinogenic. Some botanical ingredients, such as alkenylbenzenes including safrole, methyleugenol, and estragole, have been found to be toxic to human health.

#### 3.3.1. Estragole

Estragole is found naturally in several herbs, including tarragon, sweet fennel, sweet basil, anise, and star anise. It is applied as a flavor and fragrance in many food items as well as in perfumes, soaps, and detergents. Despite being approved for food application, studies have shown that estragole and its breakdown products can damage DNA and cause liver tumors in certain types of mice. Nowadays, it is inferred that estragole is a natural substance that can be both genotoxic and carcinogenic in experimental animals [119].

#### 3.3.2. Methyleugenol

Methyleugenol is a natural compound found in plants such as nutmeg, lemongrass, tarragon, pimento, basil, star anise, and fennel. Human exposure to this contaminant can occur through the consumption of food products flavored with these aromatic plants and their essential oil fractions. This natural constituent is used as a flavoring product in jellies, baked goods, chewing gums, non-alcoholic beverages, relish, and ice cream, and as a fragrance in a variety of cosmetic products [119]. There is evidence that methyleugenol and its proximate metabolite are carcinogenic, inducing liver tumors in mice and rats [119,120]. In addition, neuroendocrine tumors of the glandular stomach, renal tubular hyperplasia, and adenomas were observed at higher doses [119]. In vitro, methyleugenol and its metabolites have been found to be responsible for unscheduled DNA synthesis (UDS) in cultured rat hepatocytes [121].

#### 3.3.3. Safrole

Safrole is a natural constituent of various spices, including nutmeg, cinnamon, anise, mace, black pepper, and sweet basil. Among these, nutmeg, mace, and their essential oils are the most important dietary resources. Cola drinks can also contain safrole, and the addition of safrole to food commodities may not happen directly. Safrole was the first of the class of alkenylbenzenes to show carcinogenic characteristics [119].

### 3.4. Other Contaminants

Some other contaminants are directly or indirectly linked to our food system and may negatively affect public health. Additionally, our environment is at high risk due to the application of certain crop protection chemicals.

#### 3.4.1. Pesticides

Pesticides are toxic chemicals intentionally released into the environment to kill certain living organisms, including weeds (herbicides), insects (insecticides), fungi (fungicides), and rodents (rodenticides). Pesticides are widely used in agricultural fields, primarily by farmers, to chemically control pests. Although the purpose of pesticides is to prevent, remove, or control harmful pests, they can seriously harm both human health and the environment, including animals, air, soil, and water [122,123]. Even a very small amount of exposure can affect health during the early stages of development [124]. The risks from pesticides depend on their toxicity and exposure level. Children, pregnant women, and older adults can be more sensitive to the effects of pesticides than others [122]. Possible health effects due to pesticide exposure include cancer, asthma, allergies, hormone disruption, and hypersensitivity [122]. Moreover, there is evidence of nervous system damage, which may include peripheral neuropathy and central nervous degenerative disease, with special emphasis on Parkinson’s disease [123]. Birth defects, reduced birth weight, fetal death, etc., have also been reported due to pesticide exposure [122].

#### 3.4.2. Biogenic Amines

Usually, biogenic amines (BAs) are formed in food by the microbial decarboxylation of amino acids. Common BAs in foods include histamine, tyramine, putrescine, cadaverine, tryptamine, 2-phenylethylamine, spermine, spermidine, and agmatine. BAs are stable and difficult to degrade, as they are not destroyed during cooking, high-temperature treatment, or storage. However, the presence of BAs in food can cause adverse effects on human health. Several pharmacological and toxicological reactions may occur when consuming food products that are highly concentrated in BAs. As a result, headache, nausea, flushing, digestive problems, hypotension or hypertension, migraine, skin allergy, and heart palpitations can be observed. Histamine toxicity is known as “Scombroid Fish Poisoning,” and tyramine poisoning is called “Cheese Reaction”. In addition, many biogenic amines (BAs) may act as precursors to the formation of nitrosamines in the presence of nitrates, which have been associated with carcinogenic and mutagenic activity [125,126,127,128,129,130].

Unintentional food contaminants and associated health issues are summarized in Table 2, along with their probable sources.

## 4. Social and Economic Impacts

Undesirable chemicals in food have diverse impacts on social, economic, and environmental factors [132]. A few thousand to millions of Euros are wasted due to food adulteration [132]. Economic factors like the financial cost of analysis and monitoring, product recall and disposal, legislative cost, health costs, lost revenue and brand protection, lost productivity, and damage to the reputation of the country of origin are evaluated as assessed parameters [132]. The financial cost of analysis or monitoring is the amount associated with the sample analysis or analytical work performed during the investigation period of a chemical contamination incident. For example, the analysis cost to detect adulterations of fruit juices can have high prices, ranging from $15 to identify dilution with water to $800 to detect the presence of any pure beet sugar [66,68]. The process of removing products from distributors’ inventories, store shelves, and consumers’ homes to protect food safety is known as a food recall. Food recalls allow government agencies and companies to quickly remove potentially dangerous products from the market, helping to protect public health. Common reasons for a food recall include contamination, adulteration, misbranding, or other defects that make the product unsafe. Financial losses associated with a product recall include removal costs from the supply chain, disposal costs, repair or remediation costs, and the resulting loss of profit. For example, at the FDA’s request, Westland/Hallmark Meat Packing Company (Chino, CA, USA) recalled more than 143 million pounds of beef. The recall cost to the company was over $117 million, and in November 2012, the company agreed to a $500 million settlement with several affected customers and the federal government. Although food recalls are very expensive for companies, they are essential for maintaining and rebuilding consumer trust. Fresh spinach was contaminated with *E. coli*, triggering an outbreak across 26 U.S. states and Canada. In response, the FDA issued a recall of fresh spinach products originating from the affected region [133]. The most expensive food recall in U.S. history involved contaminated peanut products, impacting over 3900 items from at least 361 companies and resulting in an estimated $1 billion in losses for the U.S. peanut industry [134].

Health costs are considered for consumers whose health has been negatively affected by the food contamination incident, including associated medical costs. For example, in the 2008 Chinese melamine incident, free medical treatments were provided to all affected babies, with more than 1600 medical teams. It was reported that almost 300,000 children were suffering from kidney and urinary problems, and 51,900 were receiving hospital treatment [132]. Rat meat was sold as chicken in many Chinese restaurants, and this problem is also seen in England and Vietnam. Rat meat is often mistaken for chicken, and the fraudulent businessmen took advantage of this matter. These animals carry several diseases, which is a major concern associated with human health [135]. In Bangladesh, turmeric, which is a common spice in cooking, has been found as a potential source of lead exposure. High levels of lead in PRAN’s turmeric powder have been detected by local and U.S. laboratories [136]. The outbreak of E. coli contamination in fresh spinach resulted in 205 confirmed cases of infection. The outbreak led to 102 hospitalizations and three fatalities [133]. In addition, long-term consumption of contaminated food can lead to chronic health issues, resulting in frequent hospital visits. This causes significant pressure on both public and private healthcare sectors, as the rising costs of managing chronic diseases, such as diabetes and obesity caused by contaminated food, strain healthcare budgets and resources [137]. Legislative costs include civil, criminal, and regulatory costs associated with food adulteration. For example, in recent years, the magnitude of the fraud associated with individual cases prosecuted ranged from approximately $2 million to $37 million in the USA [66]. In addition, in the 2008 melamine incident in China, there were at least 60 arrests, resulting in two executions and one sentence of life imprisonment [132]. Moreover, in China, rat meat was fraudulently sold as a substitute for lamb, leading to public outrage and widespread unease about consuming meat products. The scandal resulted in the arrest of hundreds of individuals for meat-related offenses [138,139].The discovery of plasticizers in food and beverage products in Taiwan led the government to impose fines totaling approximately $40,000 on 37 companies [140]. The Italian authorities imposed fines totaling €1 million on companies involved in olive oil fraud for engaging in unfair practices. A food company in Ireland was fined £70,000 for falsely selling non-halal burgers as halal, raising significant concerns within the Muslim community [141,142].

Lost revenue or brand protection results in reputational damage to a country. The most noteworthy factor is the impact on consumers. They generally lose confidence, and after an incident, they suspect buying the product from the same company again. Sometimes people may avoid all products from the country where the food accident occurred. Therefore, manufacturers face long-term financial losses. Moreover, several food companies went bankrupt, and many employees lost their employment. For example, approximately 40% to 60% of people in China have either stopped purchasing or are unwilling to buy domestic milk products. Meanwhile, the consumption of imported milk powder has risen from 34% to 47%, driven by concerns over melamine adulteration in domestic milk. This has significantly damaged the reputation of local brands, which will require considerable time and effort to regain consumer trust [143,144]. In addition, fresh spinach contamination in the U.S. caused an estimated loss of $74 million in sales in the spinach industry [133]. Farmers and producers (such as those in dairy, coffee, honey, and wheat production) may suffer due to weak relationships in the long-chain production industry. For example, farmers faced significant losses due to a shortage of meat cows and rising feed prices during the dairy scandal in China [3].

The burden of disease (morbidity and mortality), mental trauma, consumer confidence, and cultural change are the results of social impacts. People’s confidence becomes threatened due to controversy and some overstated risks of possible health effects. Some food issues, such as meat authenticity, are a growing concern for the public as it is associated with consumers’ lifestyles (e.g., vegetarianism and organic food) and religious beliefs [145,146]. For example, the horsemeat scandal in Europe (2013), where horse DNA was discovered in processed beef products, sparked public outrage and affected consumers’ trust in the food industry [21,147]. Following the horsemeat scandal, the discovery of pork meat and porcine DNA in some processed “halal” products in the UK caused widespread panic and distress among the Muslim community, as the consumption of pork or its derivatives is strictly prohibited in Islam [141].

**Figure 3:**
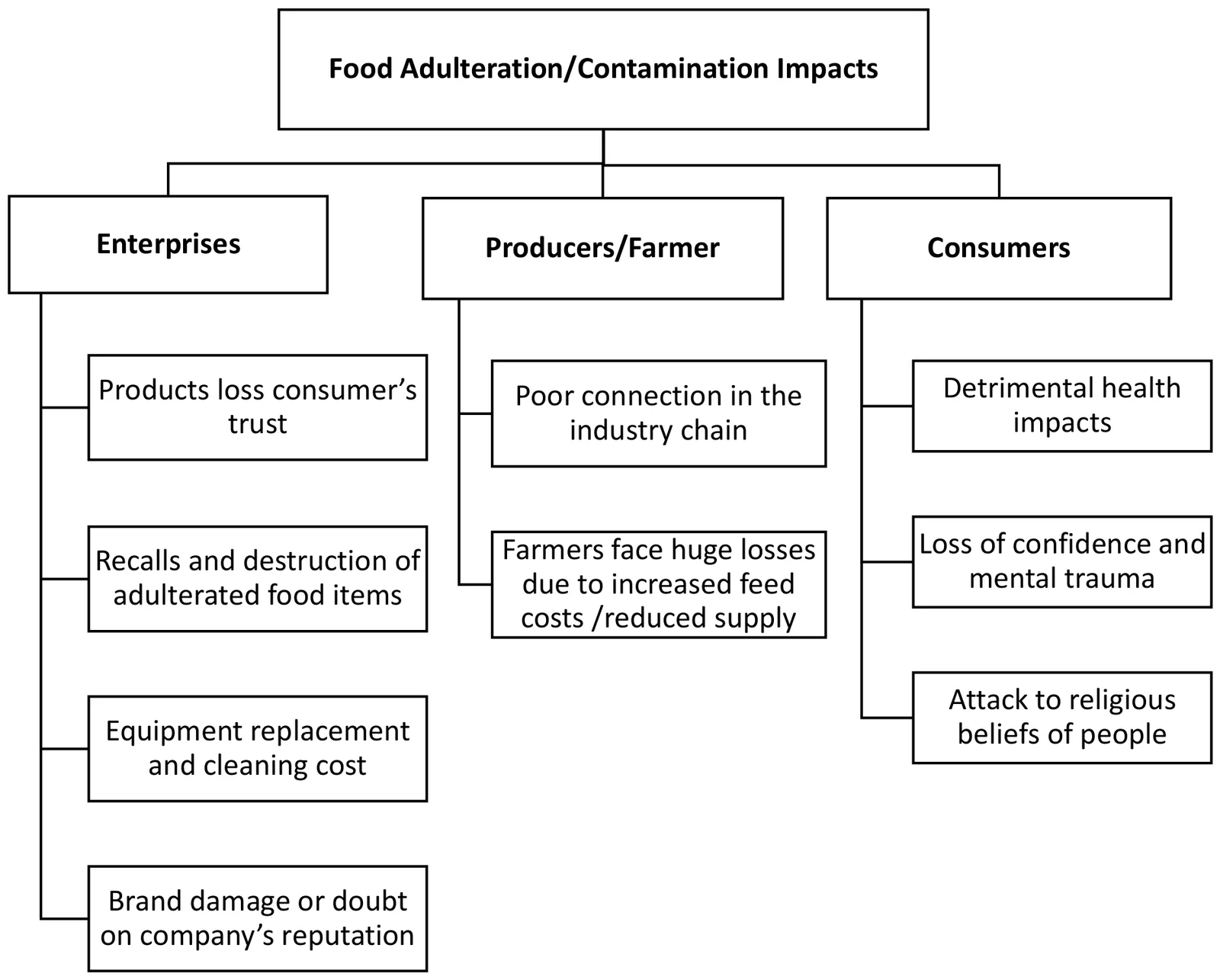
Impacts of food adulteration and contamination on different classes of people [3,144].

In summary, food adulteration has severe impacts on four types of groups: producers/farmers, processors or manufacturers/enterprises, consumers, and the government. A detailed diagram is shown in Figure 3, listing the potentially affected groups of food adulteration and contamination.

## 5. Existing Rules and Regulations

A food is considered adulterated when it does not meet the safety standards defined by the Food and Agriculture Organization (FAO) and the World Health Organization (WHO) [148]. Food industries often fail to strictly adhere to regulations and import food materials that are not always properly tested, thereby increasing the risk of unusual occurrences. In most situations, adulteration is discovered after undesirable and serious accidents take place. Hence, it is important to take firm, strict action to mitigate food adulteration and contamination incidents. Different rules and regulations are established to ensure consumer expectations, maintain food safety, address concerns about food adulteration, and respect religious beliefs regarding food. Along with global standards set by the WHO, many countries have specific laws and regulations regarding food adulteration and contamination.

### 5.1. Worldwide Standards and Organizations

The World Health Organization (WHO) works to protect and improve health worldwide. Under the International Health Regulations, the WHO requires UN member countries to report any “Public Health Event of International Concern,” which also covers unsafe food products [149].

With the establishment of the World Trade Organization (WTO) in 1995, an international treaty that includes agreements on both the Application of Sanitary and Phytosanitary Measures (SPS Agreement) and Technical Barriers to Trade (TBT) came into effect. Under this agreement, the WTO sets restrictions on member states’ policies regarding food safety inspection, along with diseases related to animal and plant health through the World Organization for Animal Health (OIE) and the International Plant Protection Convention, respectively [149].

The Codex Alimentarius Commission, part of the joint FAO/WHO food standards program, has more than 180 member countries. It is the main international reference for evaluating national food safety policies. Since 1962, Codex has created many food safety standards, guidelines, codes of practice, and recommendations. It has reviewed the safety of over 500 food additives and contaminants, and has established maximum residue limits for about 2500 pesticide/commodity combinations. The purposes of Codex are to protect consumers’ health and ensure fair international trade in food. Codex, in association with its committees, presents numerical standards, codes of practice, and other guidelines, and promotes acceptance and execution of its standards by national governments [150].

### 5.2. Legal Status: United States

In the United States, food laws prohibit two activities: adulteration and misbranding, also known as false labeling. The Pure Food and Drug Act of 1906 addresses fake food or drug advertising and foods with naturally occurring toxic substances. It allows for the inspection of food or drug warehouses. However, this law did not establish any food standards. The Food, Drug, and Cosmetic Act of 1938 is also known as one of the most important laws in food and drug regulation. This law includes new food regulations provisions that set safe tolerance limits for unavoidable poisonous substances in food. The Food and Drug Administration Modernization Act of 1997 has some provisions that include accelerating the review of devices, regulating the advertisement of unapproved uses of approved drugs and devices, and regulating health claims for foods [151,152]. Other major laws established for specific products/objectives are [153]:

- Federal Meat Inspection Act of 1906;
- Poultry Products Inspection Act of 1957 (as amended in 1968);
- Food Additive Amendment of 1958;
- Color Additive Amendment of 1960;
- Fair Packaging and Labeling Act of 1966;
- Egg Products Inspection Act of 1970;
- Dietary Supplement Health and Education Act of 1994;
- Nutrition Labeling and Education Act of 1990;
- Food Quality Protection Act of 1996;
- Food Allergy Labeling and Consumer Protection Act of 2004.

### 5.3. Legal Status: Europe

European food law establishes stringent legal standards for food products, packaging, labeling, and advertising. There are three major laws on food hygiene reformed by the European Commission: i. Regulation (EC) No. 852/2004 on the hygiene of foodstuffs; ii. Regulation (EC) No. 853/2004 on specific hygiene rules for animal-origin food; and iii. Regulation (EC) No. 854/2004, on specific rules for the organization of official controls on animal-origin products intended for human consumption [154]. Furthermore, Regulation (EC) No. 178/2002 establishes the European Food Safety Authority and outlines procedures for addressing food safety issues. Regulation (EC) No. 882/2004 establishes the official controls performed to verify compliance with feed and food law, rules on animal health, and animal welfare [10,154].

The Food Improvement Agents Package (FIAP) includes Regulations (EC) No. 1331/2008, 1332/2008, 1333/2008, and 1334/2008, which govern the use of food additives, food enzymes, food flavorings, and certain flavoring ingredients in foods. These regulations set the rules for how such substances may be used and labeled, the maximum levels allowed, and the related reporting obligations. Under EU law, food additives, flavorings, and enzymes can be marketed and added to food only after they have been approved by the European Commission and listed as authorized substances [10].

Regulation (EU) No. 1169/2011 establishes harmonized rules for food labeling. In addition, any material used for food packaging must meet strict regulatory standards. To avoid misleading advertising and to ensure that health and nutrition claims are scientifically sound, food advertising in the European Union is tightly regulated by law [10].

### 5.4. Legal Status: Middle East and Africa [154,155,156]

To address foodborne diseases, many countries in the Middle East and North Africa (MENA) region have introduced various measures. Laws have been enacted to strictly control both local and imported food products and to protect public health. Because of climate change and limited water resources, food security has become a critical concern in the MENA region. The Gulf Standardization Organization (GSO) works to harmonize Gulf Cooperation Council (GCC) standards and technical regulations for its member states (UAE, Bahrain, Saudi Arabia, Oman, Qatar, and Kuwait) in line with the Codex Alimentarius [157]. The Gulf Rapid Alert System for Food (GRASF), which connects all GCC member states, was developed by the GCC Food Safety Committee to enable regulatory action based on information on food alerts and scares. A new food safety law in Oman has been publicized to safeguard human health and safety [158]. A national food and drug authority has also been created to elevate food safety standards to international levels. Saudi Arabia treats food safety as a key public health responsibility and follows the Codex Alimentarius and other standards recognized by the World Trade Organization [158]. Lebanon passed a food safety law that applies to all types of food and beverages, as well as processed foods, and provides the regulatory requirements for food safety from farm to fork [159]. Tunisian food legislation is contained in several general laws that aim to secure the highest food hygiene level throughout the food chain [160].

Many MENA countries have limited ability to put food safety plans and policies into practice. They must balance their national food safety standards with international requirements. Several countries in the MENA region have worked hard to establish new food regulation systems and have received financial aid from various organizations, such as the U.S. Agency for International Development (USAID) and the World Bank. Examples of food safety regulatory bodies in the region include the Jordanian Food and Drug Authority (JFDA); Egypt’s Food Safety Agency Project (FSAP); Tunisia’s Agence nationale de contrôle sanitaire et environnemental des produits (ANCSEP); Morocco’s Office national de sécurité sanitaire des produits (ONSSA); the Ministry of Health in Abu Dhabi; and the Saudi Food and Drug Authority in Saudi Arabia.

Organic food production in South Africa is well known throughout the world [161]. Though there is no dedicated law for organic production, certain existing laws and policies are used to oversee the sale of organic products in the food supply chain [161]. Under the Agricultural Product Standards Act (APSA), the Biodynamic and Organic Certification Authority (BDOCA) oversees and regulates the sale of organic products. Other laws monitor agricultural food production, the sale and manufacture of food products, and fraudulent information [162].

### 5.5. Legal Status: Sub-Saharan Africa

Countries in Sub-Saharan Africa have established monitoring systems to address the growing threat of food fraud and food safety concerns. Governments have adopted international standards and have held specialized agencies responsible for inspecting food production, processing, and distribution facilities [163]. Food safety commitments are clearly outlined in public policy documents and backed by law in South Africa, Ethiopia, and Kenya. In Tanzania, food safety is addressed through the Agricultural Policy of 1997, the Livestock Policy of 2006, and the food and nutrition policy issued by the Ministry of Health in 1992 [164]. Ghana and Mozambique do not have a clearly defined, standalone food safety policy. Instead, several government agencies are authorized under different laws to address food safety concerns [165].

The National Council on Health of Nigeria formulated the National Food Safety Policy with the aim of drawing on membership from the public and private sectors relevant to the production, storage, processing, distribution, transportation, and sale of food [166]. Currently, the implementation of food safety legislation is fragmented across federal, state, and local government areas [167]. Federal agencies involved include the Federal Ministries of Health, Environment, Agriculture, Science and Technology, and Trade and Investment, as well as the National Agency for Food and Drug Administration and Control (NAFDAC), Standards Organization of Nigeria (SON), Consumer Protection Council, Nigeria Customs Service, National Biotechnology Development Agency (NABDA), Nigerian Institute of Food Science and Technology (NIFST), National Agricultural Seeds Council, and the National Biosafety Management Agency [167].

Food safety management in Ethiopia is a shared responsibility of the Ministry of Health (MoH), the Ministry of Agriculture (MoA), the Quality and Standards Authority of Ethiopia (QSAE), the Environmental Protection Authority (EPA), the Ministry of Industry (MoI), the Ministry of Trade (MoT), different federal and regional governmental bodies, research institutions, the Ministry of Education (MoE), food manufacturers, food distributors, and hotels [168,169,170]. The Ethiopian Food and Drug Authority (EFDA) is a significant body responsible for regulating the safety, quality, and efficacy of food and drugs [171]. However, there is no comprehensive food law that clearly defines the activities of these regulatory agencies [172]. Mostly, international food standard guidelines are followed to ensure food safety. In the last decade, the National Codex Committee (NCC) set Ethiopian standards through the active participation of all stakeholders [173].

The Food and Drugs Authority (FDA) of Ghana enforces the Food and Drug Act 1992 (PNDC L305B) and its 1996 amendment (Act 523), which regulates the processing, import, export, packaging, storage, transport, distribution, and sale of food. The Diseases of Animals Act, 1961 (Act 83) authorizes veterinary officers to inspect all animals intended for slaughter. The Veterinary Surgeons Law, 1992 gives veterinary doctors responsibility for treating food animals to help ensure the safety of foods of animal origin [165]. In Kenya, the stakeholders in the public sector are the Ministry of Public Health & Sanitation, the Ministry of Livestock Development (Department of Veterinary Services), the Ministry of Fisheries Development, Kenya Bureau of Standards (KEBS), Kenya Dairy Board (KDB), Kenya Meat Commission (KMC), Local Government Authorities (LGAs), and the Pest Control Products Board (PCPB). There are also actors from the private sector who primarily monitor dairy, meat, and meat product processing. Thirteen institutions in Mozambique are involved in food safety regulation and implementation, with the Department of Environmental Health at the Ministry of Health leading enforcement of laws. The main ministerial decrees compiled as food laws in 1994 are [165]:

- Ministerial Order No. 80/87 approving the hygiene regulation on food imports;
- Ministerial Order No. 88/87 approving the regulation on pesticides;
- Ministerial Order No. 51/84 approving hygiene regulations for food handling establishments.

In South Africa, several government bodies involved in food safety regulation are the Department of Health (DOH), the Department of Agriculture, Forestry and Fisheries (DAFF), and the Department of Environmental Affairs and Tourism (DEAT). The importation of consumable goods is regulated by the Foodstuffs, Cosmetics, and Disinfectants Act of 1972, and the Agricultural Products Standards Act of 1990. However, the regulatory system in South Africa appears complicated due to the complexity of supply-chain legislation [165,174]. The regional standards in Tanzania are based on the standards and guidelines established by the FAO/WHO Codex Alimentarius Commission (CAC) and the phytosanitary measures stipulated by the World Animal Health Organization (OIE) [165]. In addition, the Tanzania Food and Drugs Authority (TFDA) plays a significant role in monitoring food safety, quality, and nutritional labeling standards in Tanzania [175]. The Uganda National Bureau of Standards (UNBS), Zambia Bureau of Standards (ZABS), and Rwanda Food and Drug Authority (FDA) play a significant role in ensuring food safety and security in their respective countries.

### 5.6. Legal Status: India [176]

India’s first major national food law was the Agricultural Produce (Grading and Marking) Act of 1937. It was amended several times, with the final version issued in 1986, commonly known as the Agmark Act [177]. This Act established systems for grading and marking agricultural and related products to ensure that consumers receive quality goods. Today, the Food Safety and Standards Act (FSSA) of 2006 is the main law governing food in India, covering the manufacture, storage, distribution, sale, and import of food products and enforcing food safety and standards [178,179]. The Prevention of Food Adulteration Act, 1954 (PFA) was enacted with the primary goal of regulating sanitation, licensing, and other necessary permits required to start and run a food business. The PFA Act and its rules were amended several times. Under the 1986 version, any consumer could take food samples, have them tested, and then initiate legal action against those responsible for food adulteration [180,181]. Besides the PFA Act, other regulations were established to prevent fraud and adulteration, and these laws are presented below [182]:

- The Vegetable Oil Products Order, 1947;
- Essential Commodities Act, 1955;
- The Fruit Products Order, 1955;
- The Solvent Extracted Oil, Deoiled Meal, and Edible Flour Order, 1967;
- The Meat Food Products Order, 1973;
- The Edible Oils Packaging Order, 1988;
- The Milk and Milk Products Order, 1992.

### 5.7. Legal Status: Pakistan

Pakistan has experienced numerous food safety and adulteration issues, especially with ready-to-use food products [183]. There was no food safety legislation when Pakistan came into being in 1947. Currently, there is still a lack of a comprehensive legislative framework [183,184,185,186]. The regulatory framework is complex and fragmented, with several laws governing food safety management in the country [187]. However, several major rules govern food safety.

- The Pure Food Ordinance, 1960;
- Pakistan Hotels and Restaurants Act, 1976;
- The Pakistan Standards and Quality Control Authority (PSQCA) Act, 1996;
- Pakistan Pure Food Laws (PFL), 1963.

The establishment of food laws in Pakistan began with the Pure Food Ordinance of 1960, also called the West Pakistan Ordinance VII of 1960. This ordinance was introduced to bring together and unify laws governing the preparation and sale of food [186]. This law sets standards for coloring, flavoring compounds, antioxidants, preservatives, stabilizers, anti-caking agents, and non-nutritive constituents to prevent the presence of impurities in food. It also requires food importers, manufacturers, and retailers to ensure food safety at every stage of preparation, including manufacturing, processing, packaging, labeling, shipping, distribution, and quality control. The ordinance applied across Pakistan, except in cantonment areas, where the Cantonments Pure Food Act of 1966 was enforced instead. This act was largely similar to the Pure Food Ordinance, differing mainly in its area of jurisdiction [183,186,188]. These laws have been criticized for years for failing to provide consumer compensation in cases of harm caused by adulterated food products. This law also lacks regulations for street-vended food items, which are a common self-employment opportunity for the low- and middle-income classes in Pakistan [183,186].

The Pakistan Hotels and Restaurants Act was enacted in 1976 to monitor the standards of service and food pricing in hotels and restaurants. According to this act, the sale of contaminated food and beverages is prohibited. Consumers can address their complaints to a federal government-appointed controller regarding this issue. This act also mandates that hotel owners be licensed and conduct regular testing of food items to ensure compliance with food safety and hygiene standards. However, this act does not account for consumer compensation in the event of injury resulting from the consumption of unsafe food [183,186,189].

The Pakistan Standards and Quality Control Authority (PSQCA) was established in 1996 under the Pakistan Standards and Quality Control Authority Act to formulate standards, adopt international standards with membership from the International Organization for Standardization (ISO), and conduct product quality tests. This autonomous body became fully functional under the Ministry of Science and Technology (MoST) in 2001. It is now recognized as the National Standards Body (NSB) and is responsible for enforcing food safety and quality standards. Its services include drafting standards, providing guidance through publications and product information, inspecting food production facilities, testing food products, and issuing compliance certificates [183,186,190].

Besides national laws, there are provincial food laws set up by provincial food authorities. The Punjab Food Authority (PFA) is a notable example, having been highly active since its inception in 2011. This food authority has organized food sampling and testing campaigns across Punjab, ensuring the enforcement of food hygiene and quality standards [186,191,192].

The current legislative framework for food safety in Pakistan is built on the Pakistan Pure Food Laws (PFL), first enacted in 1963 and updated in 2007. This law underpins trade-related food quality and safety standards. It classifies 104 food items into nine main groups: beverages, food grains and cereals, starchy foods, spices and condiments, sweeteners, fruits and vegetables, dairy products, and processed foods. It also regulates food additives, preservatives, colorants, antioxidants, and heavy metals [184,185,186,193,194].

In Pakistan, the federal government oversees the import of food products. The provincial governments are responsible for enforcing food safety standards. At the national level, the federal authorities follow the standards set by the Codex Alimentarius and the U.S. FDA. However, “Halal” certification is mandatory for animal food products. The Pakistan National Accreditation Council (PNAC) is responsible for implementing the Halal Foods Act of 2016 and governs the accreditation of the Pakistan Halal Authority (PHA). The Ministry of Food Security and Research (MNFSR), established in 2011, drafts the National Agriculture and Food Security Policy and oversees federal-level food import regulations. Most functions, including agriculture, have been devolved to the provinces under Pakistan’s 18th Constitutional Amendment. Thus, provincial governments are developing their own regulations, leading to inconsistencies across Pakistan [186].

### 5.8. Legal Status: Nepal

Nepal has experienced several incidents of food fraud and contamination in recent years. The government of Nepal has developed a Food Safety Policy to ensure food safety. Several regulatory frameworks have been created to address food security [195,196].

The Food Act 1966 is the primary legislation governing food safety. This Act prevents food adulteration and contamination by prohibiting the production, sale, and distribution of inferior, contaminated, or unsafe food items. This act puts the prosecution on for misbranding food items. This act also mandates a license for food establishments. The establishment of government bodies responsible for enforcing food safety-related regulations is another provision proposed in this act. This legislation also covers the proper packaging, labeling, and storage of food items. The Food Act 1966 outlines the roles and responsibilities of the Department of Food Technology and Quality Control (DFTQC), as well as the roles of food inspectors, and provides technical specifications for quality inspection and labeling [197,198,199].

The Animal Health and Livestock Act 1998 regulates the production, sale, and distribution of animal-based food products. This act also regulates the import and export of livestock, their products, and materials essential for livestock production, including those important for food and health [195,199]. Provisions for animal quarantine posts were established to monitor the safety and quality of imported animals, animal-based food, and associated materials [197,200,201].

The Plant Protection Act 2007 and the Plant Protection Rules 2010 were derived from three earlier laws: the Food Act 1966, the Plant Protection Act 1972, and the Animal Health and Livestock Services Act 1998 (Pant, 2007). They were introduced to meet international market requirements and to comply with the Sanitary and Phytosanitary (SPS) Agreement under the World Trade Organization (WTO). These laws set standards to ensure food safety and to protect human, animal, and plant health from foreign pests and diseases [5,6]. The main goal is to protect local fauna by preventing pests from entering, becoming established, or spreading through trade in plant products. The law also covers entry permits, sanitary certificates, and re-export certificates for plants and plant products. In addition, the Pesticide Act 1991 and its 1993 Regulations control pesticide use and set maximum residue limits in agricultural products. As a WTO member, Nepal must base its sanitary and phytosanitary measures on the standards and recommendations of the Codex Alimentarius Commission, the World Organization for Animal Health (OIE), and the International Plant Protection Convention (IPPC) [202]. However, lack of adequate resources is a constraint in adopting all international standards and guidelines [203,204,205].

The Animal Slaughterhouse and Meat Inspection Act, 1999 and Regulation 2001 were established to ensure the production of healthy meat using slaughterhouses and its distribution in the Bagmati zone [197,199]. The Consumer Protection Act 1999 protects consumer rights related to the quality and safety of food. This act also includes labeling requirements. It provides for the establishment of the Consumer Protection Council to support the government in consumer-related matters [206].

The traditional food safety regulations in Nepal were based on inspecting and analyzing end products to ensure food safety. However, the focus has gradually shifted to all levels of production, processing, transportation, and trading [203]. The modern regulations were formulated in accordance with Codex principles and guidelines, focusing on preventive measures to ensure the production of safe food. Existing standards have been reviewed to ensure compliance with Codex standards, as mandated by national regulations and infrastructure [197].

### 5.9. Legal Status: Bangladesh

Bangladesh has introduced several laws, regulations, and policies to control food adulteration. These are enforced by different ministries and their respective departments. In addition, food samples are analyzed in various government laboratories [148,207]. The Bangladesh Standards and Testing Institution Ordinance of 1985, later amended by the Bangladesh Standards and Testing Institution Amendment Act of 2003, establishes an institution responsible for standardization, testing, metrology, quality control, and the grading and marking of goods. Under this law, the government established the Bangladesh Standards and Testing Institution (BSTI), which certifies the quality of commodities and materials [208].

The Bangladesh Pure Food Ordinance 1959 aims to establish better control over the manufacture and sale of food for human consumption. Generic standards for 107 food products are specified in The Bangladesh Pure Food Rules, 1967, which are currently under revision. The Food Grains Supply (Prevention of Prejudicial Activity) Ordinance of 1956 introduces special measures to prevent harmful activities. It also serves as the legal basis for preventing the spread of false information. Under the Radiation Protection Act 1987, the Institute of Food and Radiation Biology (IFRB) of the Bangladesh Atomic Energy Commission is concerned with food irradiation research and development [208]. In addition, the country has several other laws and regulations (listed below) designed to ensure safe and quality food for human consumption [148].

- The Animals Slaughter (Restriction) and Meat Control (Amendment) Ordinance, 1983;
- Destructive Insects and Pests Rules (Plant Quarantine) 1966, amended up to 1989;
- Agricultural Produce Market Act 1964 (revised in 1985);
- Fish Products (Inspection and Quality Control) Rules 1997;
- The Food or Special Courts Act 1956;
- The Pesticides Ordinance, 1971 and the Pesticide Rules, 1985;
- Bangladesh Food and Nutrition Policy 1997 and National Policy of Nutrition 1997;
- Bangladesh Food Policy 1998;
- Comprehensive Food Security Policy 2001 and New National Food Policy 2006;
- National Agriculture Policy 1999;
- Bangladesh Health Policy 2002.

### 5.10. Legal Status: Thailand

The food safety framework in Thailand is regulated by the Ministry of Agriculture and Cooperatives (MOAC), the Ministry of Public Health (MOPH), the Ministry of Commerce (MOC), the Ministry of Industry (MOI), and the Ministry of Natural Resources and Environment (MNRE) (177). Food safety systems in Thailand are divided into two operational categories [209]:

- Mandatory standards, comprising the state-enforced policies aligned with the Codex Alimentarius. It includes Good Manufacturing Practice (GMP), mandated by the Thai FDA for 54 food product categories.
- Voluntary standards, presenting discretionary benchmarks such as Hazard Analysis and Critical Control Points (HACCPs) and International Organization for Standardization 22000 (ISO 22000). Such standards are adopted by producers to access highly competitive export markets and meet elevated consumer demands.

The Food Act B.E. 2522 (1979) is the principal regulatory law for overall food safety management [210]. The Agricultural Standards Act B.E. 2551 (2008) was established with a focus on fresh produce. The 2004 Road Map of Food Safety was established to monitor supply-chain regulations [211]. The National Bureau of Agricultural Commodity and Food Standards (ACFS) coordinates the agricultural regulations with international benchmarks. Thailand also reestablished the “Thai Food-to-the-World Project” to maintain its reputation as the “kitchen of the world”. With an aim to enhance food security, Thailand started to collaborate with neighboring nations in 2009 by adopting the ASEAN Integrated Food Security (AIFS) Framework and the Strategic Plan of Action on Food Security (SPA-FS) [211].

As a local effort, the Food Sanitation Division of Bangkok Metropolitan Administration (BMA) actively monitors vendors and markets to align with central health policies. To maintain transparency with the public and other sectors, the Food Sanitation Division compiles data from all fifty districts to produce a yearly food safety report. Moreover, Thailand also adopted a “From-Farm-To-Table” policy to combat physical, chemical, and biological hazards in food products [212].

### 5.11. Legal Status: China

The food safety regulations in China were dominated by the Department of Light Industry in the early 1950s. However, multiple safety incidents resulted from inadequate technological support and poor hygiene practices [213]. The 1982 Food Hygiene Law was established to cope with the rapid economic growth. In the early 2000s, food safety monitoring was not confined into a single agency. Regulations were distributed across different sectors, including agriculture, health, and industry. Following the jurisdictional issues arising from multiple regulatory authority, the food safety regulation was centralized. The China Food and Drug Administration (CFDA) took over the supply-chain responsibility in 2013, followed by a 2018 integration into the State Administration for Market Regulation for comprehensive oversight. Despite centralization efforts, specific roles remain delegated among key government bodies [214]:

- The Food Safety Committee of the State Council provides overall food safety guidance.
- The National Health and Family Planning Commission formulates national food safety standards.
- The Ministry of Agriculture supervises the planting, breeding, and slaughter stages of agricultural products.
- China Food and Drug Administration (CFDA) supervises food from processing to the consumer’s table.

According to the 2009 Food Safety Law, the government regulates the industry, and companies bear the primary responsibility for food safety. This policy resulted in better inspection management. Food Traceability Systems (FTSs) were also initiated in 2004, backed by 52 national laws and 118 local regulations that promote technology and investment requirements. As part of its implementation, early vegetable tracking was established in Beijing in 2004. Advanced RFID and GPS tracking technologies were used for food safety monitoring during the 2008 Olympics. In 2010, the Chinese government invested 1.86 billion yuan to trace meat and vegetables across 50 cities. The national FTS platform was tracking over 34 million items by late 2014 [215].

### 5.12. Legal Status: Japan

Scarcity of agricultural food products has always been a major concern in Japan due to frequent natural disasters. The economic boom in 1960 shifted the national diet from traditional staple foods to red meat, dairy, and processed foods. The dependency on processed foods resulted in several food safety incidents such as Morinaga Milk (1955), Minamata (1953), Niigata (1964), and Kanemi Rice Oil (1968). Global trade issues regarding the safety of imported food also resulted in consumer anxiety. All these vulnerabilities in food-production chain fueled Japan’s modern food safety movement. In the 1960s and 1970s, the Japanese food safety movement focused heavily on protesting state and industry policies. By the 1980s, consumers started to form small organizations to build safe agricultural systems and preserve traditional Japanese diets. The consumer cooperatives experienced substantial organizational growth by introducing guaranteed food safety in 1973. However, during the 1990s, mainstream corporations and supermarkets started to dominate the food market [216,217,218].

To reinforce food safety, the Japanese government amended the Food Sanitation Law and enacted the new Food Safety Basic Law in July 2003 [219]. The Food Safety Commission was established as an independent advisory committee under the Cabinet Office. The main roles of the commission include conducting risk assessments, promoting risk communication, and responding to emergency situations. The commission advises the Prime Minister on basic matters concerning food safety policies. It also coordinates with the Health, Labor and Welfare, and Agriculture, Forestry and Fisheries ministries [220,221].

Food imports and distribution in Japan are heavily regulated by mandatory labeling laws, including the following [220]:

**Plant Protection Law:** This law is implemented by the Ministry of Agriculture, Forestry, and Fisheries. It manages plant quarantines to screen fruits and vegetables during entry. Prohibited items may sometimes be imported if they undergo specific treatments, such as fumigation, vapor heat treatment, etc. In addition, all products must be accompanied by a phytosanitary certificate.

**Food Sanitation Law:** This law is administered by the Ministry of Health, Labor, and Welfare. It aims to prevent health hazards by imposing strict inspections for pathogens, fungal toxins, and additives. In May 2006, Japan introduced a positive list that set maximum residue levels for specific pesticides. Products exceeding these limits are not allowed to enter the country.

**Product Liability Law:** Under this law, producers and importers bear the liability for product defects. In cases of food poisoning, manufacturers face massive financial losses. It also results in product withdrawals, victim compensation, and severe damage to their brand reputation.

Moreover, inspections related to the Food Sanitation Law, including document inspection, food quality monitoring, and inspection ordered by the Ministry of Health, Labor and Welfare for inspections by importers at the time of importation are also conducted [189].

### 5.13. Legal Status: Australia and New Zealand

Food laws in Australia originated locally in Victoria and New South Wales during the 19th and early 20th centuries. Beginning in 1995, Australia shifted its focus toward performance- and outcome-based regulations. The previous food safety management system was overly complex and costly for businesses. As a result, governments adopted a framework based on a partnership between consumers, industry, and the government. Moreover, the founding of national advisement, which began in 1936, resulted in the establishment of the current dual national regulatory body, Food Standards Australia New Zealand (FSANZ), in 2000. Australia and New Zealand managed their food safety jointly through a cooperative Food Treaty and the Australia New Zealand Food Standards Code. This aimed to harmonize regulations and reduce trade barriers throughout the supply chain. An intergovernmental agreement resulted in all states and territories adopting the Food Standards Code into their own legislative frameworks. However, loopholes in trade agreements emerged from the shared system [61]. Despite the agreement with New Zealand, Australian food regulation remains fragmented across three tiers of government: Federal Government, State or Territory Governments, and Local Councils. The Food Standards Code provide uniform national food safety laws. A guide called Safe Food Australia supports the mandatory standards [61,222]:

- Standard 3.1.1 defines terminology and outlines general compliance rules for businesses and handlers.
- Standard 3.2.1 focuses on hazard control. It is only mandatory for identified high-risk industry sectors.
- Standard 3.2.2 dictates specific controls for receiving, storing, processing, packaging, and transporting food.
- Standard 3.2.3 sets the physical requirements for food premises, fixtures, equipment, and transport vehicles.
- Standard 3.3.1 requires businesses serving vulnerable populations to implement documented and audited food safety programs.

The food legislation in Australia is administered at the state level. Offenses are mostly dealt with by the administration. The administrative approach uses a tiered progression that starts with warnings and uses prosecution only as a last resort. Criminal charges for food fraud carry severe consequences. For instance, following the 2018 Australian strawberry scandal, the maximum penalty for intentional food contamination was increased from 10 to 15 years of imprisonment. The Australian Competition and Consumer Commission (ACCC) typically investigates these cases. Regulatory action heavily relies on complaints from consumers or public health campaigners [223,224].

### 5.14. Legal Status: Brazil

Food safety regulations in Brazil were dominated by religion and culture in the past. The government had a policy for penalizing the trade of adulterated goods. The first basic food standards were established in 1969. The rise of several food safety incidents due to increased food production and poor supply-chain management led to the shift toward modern regulation [225]. In the 1990s, sanitary guidelines were aligned with the international Codex Alimentarius through Administrative Rule number 1428. As a member of the Codex Alimentarius Commission, Brazil implements federal and regional regulations that align with Codex and World Health Organization (WHO) guidelines. This rule established the principles of Good Manufacturing Practices (GMP) and the Hazard Analysis and Critical Control Points (HACCPs) program [226,227,228]. The significant legislative changes in the early 2000s spread awareness among entrepreneurs and food handlers [226]. Food safety regulations in Brazil also align with the Common Market of the South (MERCOSUR) guidelines [229].

Brazil manages its food safety and quality through a comprehensive regulatory system. The regulations are operated primarily at the federal level. Additional regulations are enforced at regional levels. The federal oversight of food control is jointly handled by the Ministry of Agriculture, Livestock and Food Supply (MAPA) and the Ministry of Health. The Ministry of Health executes its food safety responsibilities through the National Health Surveillance Agency (ANVISA). The Center of Health Surveillance in Sao Paulo established the technical parameters for sanitary-hygiene control in March 1999. This rule served as a model for food control nationwide and was amended later in 2008 [229,230].

Prior to Sao Paulo’s state legislation, the federal Ministry of Health implemented general GMP requirements for food manufacturers and industries in July 1997. In October 2002, continuous control of GMPs and four Standard Operating Procedures (SOPs), including equipment sanitation, pest control, water tank sanitation, and handler hygiene were mandated. In September 2004, the Brazilian Health Surveillance Agency (ANVISA) published a Technical Regulation of Good Practices specifically for food services. This policy gave establishments 180 days to comply and required the implementation of a Good Practices manual. The federal legislation allows for supplementation by state and municipal authorities. For example, Rio Grande do Sul published Administrative Rule number 542 in 2006 to approve a Good Practices checklist and regulate training courses for handlers. Later, this rule was amended in 2009 and expanded the scope of the federal legislation to include venues such as street vendors, mini-markets, and school kitchens. This rule also specified detailed procedures for tasks such as cleaning cloths and handling raw eggs.

### 5.15. Legal Status: Argentina

Argentina is a member of the Codex Alimentarius Commission and Mercosur. The country has also been a member of the World Trade Organization (WTO) since 1995 [231]. The Argentine Food Code, called Codigo Alimentario Argentino (CAA), serves as the primary regulatory framework for local food production. The CAA is regularly updated to incorporate standards from Mercosur. Its responsibility is to protect public health and maintain confidence in commercial transactions. The principal regulatory authorities are the Ministry of Health and the National Administration of Drugs, Foods, and Medical Technology (ANMAT). Established in 1992, ANMAT is a decentralized body responsible for protecting human health. It ensures the quality and safety of foods, medicines, and medical devices across the country. The National Committee of Food (CONAL) acts as the primary scientific advisory body. CONAL supports the National Food Inspection System (SNCA). It also reviews petitions to incorporate new ingredients, additives, and processing aids into the Argentine Food Code [232].

The Argentine Food Code defines ingredients for safe addition to food products [232,233]:

**Food ingredients are** defined by Mercosur as any substances, including additives, used in the preparation of food and present in the final product. Currently, the CAA does not officially define ‘novel foods’, though they are recognized in practice.

**Direct food additives** must provide a technical effect without the intent to provide nutritional value and are regulated by Mercosur positive and negative lists. Unlisted additives must be submitted to CONAL for evaluation and subsequent Mercosur incorporation.

**Food contact substances** are defined as primary containers or wrappings in direct contact with food. There is a positive list of approved additives for use in plastics.

**Flavoring agents** are a designated list of permitted natural or synthetic flavoring agents and colorants.

**Enzymes** are defined as substances of animal, plant, or microbial origin that promote desirable chemical reactions.

**Processing aids** are defined as substances intentionally used for a technological purpose during processing. These are not consumed as ingredients and must be removed or inactivated.

**Nanoscale materials:** There are currently no authoritative regulations for nanoscale materials in food. Local working groups are developing standard methodologies for risk assessment.

## 6. Critical Synthesis and Future Recommendations

The global food laws are being actively modernized to meet the demands of international trade blocs. However, in many developing countries, domestic food safety systems still lack effective central planning and coordination for public health, creating a system of social exclusion. Certified food products remain accessible only to a small proportion of the population, while most consumers lack access to foods that meet minimum safety standards. Globally, the private sector has rapidly established stringent food standards to differentiate products and ensure quality. However, private standards are heavily imposed by buyers and retailers, driven by their requirements. Those standards are designed to force suppliers to make substantial financial investments in new equipment, training, and reporting, which often significantly reduces profit margins. Those standards are designed to force suppliers to make substantial financial investments in new equipment, training, and reporting, which often significantly reduces profit margins. Current food policies remain largely reactive, rather than preventive, to food adulteration and contamination. Significant conflict of competence among authorities due to overlapping regulations is also common in the global food industry. In addition, unauthorized use of non-authorized pesticides and veterinary antibiotics is still widely practiced worldwide due to high production costs, local availability, and lack of education and awareness at the manufacturing end. Based on the challenges identified, the following recommendations are proposed to enhance food safety and regulatory compliance:

**Food policy reforms:** It is necessary to implement a single, centralized public policy worldwide to eliminate regulatory duplication. Food regulations must be updated to account for new technologies and emerging hazards. A shift toward preventing contamination is necessary rather than merely responding to it after the fact. In addition, addressing the challenges related to capital access, administration, and incentive provision is necessary to scale private standards into public systems.

**Food quality monitoring:** Major investments are needed to expand food monitoring programs. It is also equally important to implement penalties for violations of food laws. Moreover, surveillance actions must be intensified on the use of artificial food additives and unauthorized agricultural chemicals.

**Capacity building and producer support:** The local producers should be properly trained in standard food-handling procedures. Affordable technology should be introduced into the food-production chain to meet strict safety requirements while remaining within the means of local producers. Moreover, the food industry should actively promote natural food additives as replacements for artificial substances.

**Future research directions:** Systematic research is needed on how exporting countries adjust their regulatory frameworks in response to international import restrictions. It is also essential to study global power shifts in the overall economy. International regulatory governance should be analyzed through the lens of policy learning. Further research should be done to expand food monitoring capabilities.

## 7. Conclusions

The food supply chain is a vast system that promotes adulteration and contamination. Strict monitoring and caution at each level can reduce the risk to some extent. Ignorance of food adulteration can severely impact any nation if left unchecked. The health impacts range from mild to severe. The effects can be either short-term or long-term. The economic losses are also a serious concern for any country. It would take a long time for a company susceptible to adulteration to regain its lost reputation, as public confidence is eroded by dishonest food sellers. To ensure food safety and security, it is essential to pay close attention to every sector of the food supply chain. Shifting toward a centralized public policy can eliminate regulatory duplication. The analytical techniques for contaminant detection should be modernized to ensure food security. Future research should be conducted on expanding food monitoring capabilities. Proper guidelines should also be implemented, and proper knowledge should be disseminated to prevent food adulteration. Consumers must be aware of the expiration dates, ingredients, labeling, and other relevant details when purchasing their food products. In short, food adulteration is a complex issue that cannot be addressed solely by policymakers and regulators. Food producers, vendors, and consumers all need to play a role in making their country a safe place to live.

## Figures and Tables

**Table 1:** Intentional food adulterants and possible health issues.

Adulterants	Found In/Sources	Health Effects
**Ripening Agents**		
Calcium Carbide	Fruits and Vegetables.	Carcinogenic; headache, dizziness, mood disturbances, sleepiness, mental confusion, seizures, memory loss, cerebral edema, abdominal pain, vomiting, diarrhea, difficulty in swallowing and speech, permanent skin damage, etc. [22,25,30].
Ethylene Glycol	Fruits like Bananas, Mangos, Nectarines, Apples, Lemons, etc. [27].	Kidney failure [27].
**Artificial Sweetening Products**		
Acesulfame-K	Baked goods, candies, frozen desserts, beverages, breath mints, and cough drops [34].	Clastogenic and genotoxic at high doses and headache [34].
Aspartame	Tabletop sweetener, gum, breakfast cereal and other dry products [32].	Headache, dry mouth, nausea, vomiting, dizziness, reduced seizure threshold and thrombocytopenia [34].
Saccharin	Baked goods, soft drinks, tabletop sweetener, jams, and chewing gum, etc. [34].	DNA damage, cancer, nausea, vomiting, and diarrhea [32,34,36].
**Artificial Coloring Products**		
Malachite Green	Green vegetables (green chili, green peas, bitter gourd, lady finger, and pointed gourd), fish [36,95].	Genotoxic, carcinogenic, multi-organ tissue injury, adverse effects on the immune system, and reproductive system [41,42,43].
Metanil Yellow	Sweat meat, ice-creams, soft drinks, turmeric powder, and beverages [39].	Problems in the heart, liver, kidneys, intestines, gastric tissue, nervous tissue, etc. [38].
Sunset Yellow	Sweets, jams and jellies, soft drinks, candies, canned juice, sauces, pickles, ice cream, etc. [44,45].	Mutagenicity, allergic reaction, gastric upset, diarrhea, vomiting, migraines, and hyperactivity [44,45,46].
Copper Sulfate	Green vegetables (bitter gourd, ladyfingers, and pointed gourd) [36].	Anemia [36].
Orange II	Chilli, safflower, sumac, paprika [20].	Mainly genotoxic and in some cases carcinogenic [20].
Rhodamine B	Sumac, chilli powder, curry, paprika, turmeric [20,59].	Genotoxic and carcinogenic [20,59].
Yellow 5	Candies, cereals, and baked products [36].	Hypersensitivity, hyperactivity, and other behavioral effects [36].
Blue 2	Confectionery, candies, pet foods, beverages, etc. [36].	Brain tumors [36].
Methanol Yellow	Melons and watermelons [36].	Stomach diseases, cancer, and deterioration of male reproductive organs [36].
Lead Chromate	Melons and watermelons [36].	Brain injury, blindness, and anemia [36].
Sudan Dyes (I, IV) and Para Red	Hot chili, other spices (cayenne pepper, sumac, turmeric, chili, paprika, curry), baked foods, seafood, eggs, melons, and watermelons [20,36,47,55,56,57,58].	Carcinogenic, mutagenic, genotoxic, pigmented contact dermatitis, stomach problems [20,36,47,48,50,51,52,53,54].
**Other Adulterants**		
Melamine	Milk and animal feed products.	Renal failure, kidney stones, urinary tract effects, and death in infants [91,93,94].
Growth Hormones	Vegetables [36].	uterine cancer, exhaustion and loss of energy, early or irregular periods in women, balding in men, etc. [36].
Formaldehyde (FA)	Fish, fruits, milk, cheese, vegetables, drinks, sweetmeat, ice-cream, and spices [86,87,88].	Problems in nervous system, kidney, liver and lungs; asthma, pulmonary damage, abdominal pain, vomiting, nausea, gastrointestinal lesions, nasopharyngeal cancer, gastrointestinal cancer, leukemia, irritation on body parts, metabolic acidosis, proteinuria, hematuria, tachypnoea, jaundice, reduced blood cells, acute renal failure, DNA and chromosomal damage [86,87,88,89,90].

**Table 2:** Incidental food contaminants and possible health hazards.

**Trace Elements**		
Aluminum (Al)	Food, drinking water, and beverages.	Neurotoxic, brain disease, bone softening, anemia, Alzheimer’s disease [100,101,102].
Arsenic (As)		Blackfoot disease, lung, skin, and other internal cancers, skin lesions, peripheral neuropathy, and anemia [104,105].
Cadmium (Cd)		Lung and prostate cancer, reduced cognitive development and intellectual performance, problems in the kidneys and the reproductive system, hypertension, increased blood pressure, renal toxicity [97,104,106].
Chromium (Cr)		Carcinogenic, genotoxic, kidney, liver damage, skin lesions or rashes [108].
Fluoride (F)		Dental and skeletal fluorosis [112].
Lead (Pb)		Decreased cognitive development and intellectual performance, affected kidneys and the reproductive system, and hypertension [97,99,104].
Manganese (Mn)		Neurological effects [98].
Copper (Cu)		Excessive salivation, epigastric pain, diarrhea, nausea, vomiting, abdominal pain, intravascular hemolytic anemia, acute tubular renal failure, acute hepatic failure, coma, shock, and death [110,111].
**Mycotoxins**		
Aflatoxins	Maize, rice, wheat, peanuts, tree nuts and dried fruits [114,115].	Immunosuppressive, teratogenic, mutagenic, and carcinogenic [116].
Ochratoxins	Cereals, bread, wine, coffee, spices and dried fruits [115,116].	Carcinogenic, teratogenic, immunosuppressive, nephrotoxic, and urinary tract tumors [115,116].
Trichothecenes	Cereals and cereal products [114].	Depression of immune responses, nausea and vomiting [116].
Fumonisins	Maize and maize based products [115].	Carcinogenic [115].
Patulin	Apples, apple juice [114].	Damage of gastrointestinal, respiratory systems, DNA, many enzymes etc. [114].
Zearalenone	Wheat, maize, barley [116].	Premature puberty in girls, cervical cancer [114].
**Natural Toxins**		
Estragole	Herbs like tarragon, sweet fennel, sweet basil, anise and star anise [119].	Genotoxic and carcinogenic [119].
Methyleugenol	Natural ingredient of nutmeg, lemongrass, tarragon, pimento, basil, star anise, fennel and flavoring agent in jellies, baked goods, chewing gums, non-alcoholic beverages, relish and ice cream [119].	Carcinogenic and unscheduled DNA synthesis (UDS) [119,121].
Safrole	Natural constituent of nutmeg, cinnamon, anise, mace, black pepper, sweet basil and present in cola drinks [119].	Carcinogenic [119].
**Other Contaminants**		
Pesticides	Agricultural fields.	Cancer, asthma, allergies, hormone disruption, hypersensitivity, nervous system damage, birth defects, reduced birth weight, fetal death, etc. [122,123].
Biogenic Amines (BAs)	Fish, meat, dairy products (particularly cheese), wine, beer, and vegetables including sauerkraut, broad bean, banana peel, and avocado [125,126,128,131].	Headache, nausea, hypo- or hypertension, flushing, migraine, skin allergy, digestive problems and heart palpitations [125,126,127,128].

## Data Availability

No new data were created or analyzed in this study. Data sharing is not applicable to this article.
